# Executive function at baseline and follow-up in opioid maintenance patients and its relation to psychiatric comorbidity and substance use patterns

**DOI:** 10.1186/s12888-025-06524-w

**Published:** 2025-04-17

**Authors:** John Hanson Högberg, Björn Philips, Jonas Nielsen, Stina Richter Simsek, Kristina Berglund

**Affiliations:** 1 Department of Psychology, University of Gothenburg, Gothenburg, 405 30 Sweden; 2 Department of Addiction Disorders, Sahlgrenska University Hospital, Gothenburg, Region Västra Götaland Sweden; 3 Department of Psychology, Stockholm University, Stockholm, 106 91 Sweden

**Keywords:** Opioid maintenance treatment, Executive functions, Opioid use disorder, Longitudinal

## Abstract

**Background:**

Research investigating executive functions in opioid-dependent patients undergoing opioid maintenance treatment (OMT) is scarce. This study aimed to assess executive function in patients with opioid use disorder at treatment initiation and one-year follow-up, exploring its correlation with psychiatric comorbidity within the patient group. Additionally, associations between executive functions and factors such as age at substance use initiation, duration of substance use, and current substance use were explored.

**Methods:**

Forty-nine adults (mean age: 40.6 [11.4]) with opioid use disorder initiating OMT participated in a naturalistic study with a one-year follow up. Participants underwent subtests of the Delis-Kaplan Executive Function System (D-KEFS) and self-assessed their cognitive function using the BRIEF-A form. Psychiatric diagnoses were determined using MINI, while symptoms of personality disorders were assessed using the SCID II screening form. Blood and saliva samples were collected for alcohol and drug markers.

**Results:**

Most participants exhibited impaired Cognitive flexibility (67%), with varying levels of impairment in Verbal fluency and problem-solving functions (25–30%). The majority rated their executive functions as poor. At the one-year follow-up, Verbal fluency had improved (*p* <.05), but other executive functions remained unchanged. Stimulant use was associated with reduced Verbal fluency and Cognitive flexibility (*p* <.1). Older age and longer substance use duration correlated with poorer Verbal fluency (*p* <.05), while earlier onset of substance use correlated with poorer self-reported executive functioning (*p* <.05) but unexpectedly with better Cognitive flexibility (*p* <.1). Symptoms of borderline personality disorder was related to poorer self-reported executive functioning (*p* <.001), symptoms of Narcissistic Personality Disorder was related to poorer Cognitive flexibility (*p* <.1), and symptoms of Antisocial Personality Disorder was related to better problem-solving (*p* <.1).

**Conclusions:**

Executive function impairment is common in patients starting OMT, with specific functions more affected. The varied results of correlations between psychiatric comorbidity, substance use, and executive function indicate patient heterogeneity. While some executive functions show slight improvement over time, complex functions appear resistant to change, suggesting lasting damage which may influence treatment outcomes. Overall, patient variability in executive function highlights the need for personalized treatment approaches in OMT.

**Supplementary Information:**

The online version contains supplementary material available at 10.1186/s12888-025-06524-w.

## Background

Opioid addiction is a global epidemic. In 2021, an estimated 60 million people used illicit opioids globally [1]. In 2019, opioids accounted for over 89,000 deaths globally. Fatal opioid overdoses were estimated to be over 80,000 in the United States alone in 2021 [2]. Approximately one million Europeans used heroin or other illicit opioids in 2021. Opioids were not among the most commonly used drugs but accounted for the largest proportion of harm illustrated by the presence of opioids in about three-quarters of fatal overdoses in the EU in 2020 [3, 4].

Opioid maintenance treatment (OMT) is the gold standard intervention for patients with opioid use disorder (OUD). OMT includes the use of therapeutic drugs, most commonly methadone or buprenorphine but also a range of psychosocial interventions [5]. OMT has shown efficacy for recovery in terms of abstinence from opioids, mortality risk, and improved overall health of patients suffering from opioid addiction [6]. However, a significant proportion of patients do not stop side use of drugs and drop out of treatment, with increased risk of mortality. OMT has showed a retention rate of less than 50% after 6 months of treatment [6]. The risk for dropout can vary between treatment periods, implying changing needs of support and additional treatment throughout the treatment process [7].

One consistent risk factor for dropout, relapse, and less successful treatment retention in patients with OUD is impaired executive function [8]. Executive function is an umbrella term used to describe a range of mental functions involved in goal-directed behaviors, including ability of cognitive flexibility, semantic retrieval, verbal fluency, visual scanning, number and letter sequencing, motor speed, hypothesis testing, abstract thinking, object recognition, visual discrimination, inhibition, and problem solving [9, 10]. When these are compromised, patients may become apathetic, restless, or impulsive [11]. A general measure of executive function is not possible to obtain through a single psychological test. Rather, a battery of tests can measure one or more function(s) [12].

Despite executive function having been shown to have a significant impact on the treatment outcomes of Opioid Use Disorder (OUD) [8], there are few studies that have examined this in patients initiating Opioid Maintenance Treatment (OMT). So far, there are three American studies that investigated cognitive function at the initiation of treatment [5, 13, 14]. The study by Mintzer et al., [14] had a narrow aim investigating dose-related buprenorphine effects on cognitive performance with a small sample involving eight participants.

In the study by Arias et al., [13], it was found that 39% of the patients had impaired neurocognitive function. The study also examined the presence of a diagnosis of depression and whether it was more common for patients with a depression diagnosis to have impaired neurocognitive function. Furthermore, it investigated whether patients with previously diagnosed alcohol dependence and cocaine dependence had poorer neurocognitive function. The results showed that lifetime alcohol and cocaine dependence was related to poorer neurocognitive function, while a diagnosis of depression was not [13].

The study by Scott et al. [5] (using the same method as Arias et al., 13) also examined how a diagnosis of depression was related to neurocognitive function. This study demonstrated that, contrary to the results from Arias et al. [13], patients with depression had higher levels of impaired neurocognitive function. Additionally, lifetime diagnosis of alcohol use disorder was not linked to impaired neurocognitive function and lifetime cannabis disorder and lifetime cocaine disorder was linked to *better* neurocognitive function.

The three studies that investigated cognitive/executive function in patients starting OMT have not explored other psychiatric diagnoses beyond depression. They also did not investigate the prevalence of personality disorders, nor did they consider the patients’ duration of drug use, factors that previous studies have shown to affect cognitive/executive function [11, 15].

There are very few longitudinal studies of executive function in OMT. Rapeli et al. reported three studies before 2014 [16]. In Rapeli et al.‘s 2011 study [17] of opioid-dependent patients treated with buprenorphine/naloxone and benzodiazepines, worse performance in working and verbal memory was reported compared to healthy controls at six months of treatment. Another study assessed changes in neurocognitive function in 17 patients after 6 months of buprenorphine-naloxone treatment for opioid dependence. It reported improvements in cognitive flexibility, decision making, attention, working memory, and psychomotor speed. The study also noted few differences in performance compared to a healthy control group after 6 months. However, excluded patients in this study included those with other substance dependence and severe mental illness, making this sample less comparable to the present sample [18].

As previous research have yielded mixed results regarding the relationship between executive function and substance use and psychiatric comorbidity in OUD patients entering treatment, this highlights the importance of more studies of cognitive/executive function. The global opioid epidemic affects millions of heterogeneous patients warranting a need to know more about the patients’ characteristics [6].

## Methods

### Aim

This naturalistic study aimed to examine the executive function in patients with opioid use disorder at the onset of treatment and at a one-year follow-up and its relationship with the most prevalent psychiatric diagnoses and personality disorders within the patient group. Additionally, we sought to investigate how factors such as sex, age at substance use initiation, years of substance use, and current substance use at the start of treatment were associated with executive functions.

### Participants

Participants were 49 patients starting OMT, diagnosed with opioid dependence (12 women and 37 men) with a mean age of 40.6 years [SD = 11.4]; range: 23–66 years). The mean years of duration of drug use was 24.7 (SD = 11.0). The range of duration of drug use was 6 to 48 years. The mean age of debut of substance use was 15.7 (SD = 3.3). The range of debut of substance use was 10 to 30 years. A significant difference was detected between male and female participants in age at onset of substance use (*U* = 126.0, *p* <.05). Females were significantly older than males at the time of onset of substance use (median 17 years vs. median 15 years). No other demographic differences were detected. Baseline demographic variables stratified by sex (for example living and social conditions, employment and education) for participants are summarized in Table 1.

**Table 1:** Background characteristics of participants (*n* = 49) data is presented as *m* (*SD)*,* mdn* or *n* (%)

	Total *n* = 49	Males *n* = 37	Females *n* = 12
**Age** (range 23–66 years)	40.6 (11.4) *Mdn*: 38	41.1 (11.85) *Mdn*: 41	39.0 (10.4) *Mdn*: 34
**Birth country**			
Sweden	41 (83.7%)	31 (83.8%)	10 (83.3%)
Other	8 (16.3%)	6 (16.2%)	2 (16.7%)
**Residence**			
Private lease	20 (40.8%)	14 (37.8%)	6 (50%)
Parent(s)’ apartment	10 (20.4%)	8 (21.6%)	2 (16.7%)
Social services	10 (20.4%)	8 (21.6%)	2 (16.7%)
Support apartment	5 (10.2%)	4 (10.8%)	1 (8.3%)
Homeless	2 (4.1%)	1 (2.7%)	1 (8.3%)
Other	2 (4.1%)	2 (5.4%)	0 (0%)
**Education**			
Elementary school	20 (40.8%)	16 (43.2%)	4 (33.3%)
High school	22 (44.9%)	16 (43.2%)	6 (50%)
University	7 (14.3%)	5 (13.5%)	2 (16.7%)
**Employment**			
Full time (work)	5 (10.2%)	3 (8.1%)	2 (16.7%)
Part time	3 (6.1%)	2 (5.4%)	1 (8.3%)
Subsidized wage comp.	14 (28.6%)	11 (29.7%)	3 (25.0%)
Pension	2 (4.1%)	2 (5.4%)	0 (0%)
Unemployed	23 (46.9%)	17 (45.9%)	6 (50.0%)
Institutionalized	2 (4.1%)	2 (5.4%)	0 (0%)
**Social conditions**			
Married	3 (6%)	3 (8.1%)	0 (0%)
Cohabiting	6 (12%)	4 (10.8%)	2 (16.7%)
Single	40 (76.7%)	37 (81.0%)	10 (83.3%)
**Children**	22 (44.9%)	16 (43.2%)	6 (50%)
**Age at onset of substance use** (range 10–30 years) **Duration of years of substance use** (range 6 − 48 years)	15.7 (3.3) *Mdn*: 15.5 24.7(11.0) *Mdn*: 23.5	15.3 (3.4) *Mdn*: 15 25.5 (11.5) *Mdn*: 24.5	16.9 (2.6) *Mdn*: 17* 22.1 (9.0) *Mdn*: 20

* *p* <.05 Mann Whitney U: 126.0

### Measures

An excerpt of questions from the Addiction Severity Index [19] was used to obtain demographic information, including age at onset of drug use. Current drug use was monitored using liquid chromatography–high resolution mass spectrometry (LC-HRMS) analysis of oral fluid and alcohol marker B-Peth for analysis of blood samples [20]. Executive function was assessed through a neuropsychological test battery using three tests from the Delis-Kaplan Executive Function System, D-KEFS. The D-KEFS has demonstrated moderate to good reliability (0.50–0.80) and has shown reasonable validity in distinguishing between control groups and clinical populations (alcoholism, schizophrenia and brain lesions) [10].

The D-KEFS *Verbal fluency Test (VFT)* measures the ability of Verbal fluency switching and production. The test provides a measure of the participant’s ability of switching between different categories, speed in semantic retrieval, and flow of verbal production. The participant’s task is to verbally produce as many words as possible while alternating between two different categories for one minute. Outcome is measured in number of correct switches between categories (switching) and correct words (production).

The D-KEFS *Trail Making Test B* (TMT) measures the ability of Cognitive flexibility based on four basic functions: visual scanning, number sequencing, letter sequencing, and motor speed. The participant’s task is to draw a line as quickly as possible between numbers and letters in alternating order. Outcome is measured in completion time.

The D-KEFS *Twenty Questions* measures the ability of Problem solving. The test examines whether there are difficulties with basic cognitive functions such as perception, object recognition, visual discrimination, and inhibition. It also shows impairments in the ability to assimilate oral feedback and Problem solving. The test participant’s task is to find out through as few questions as possible which figure the test leader chose from an image gallery. Outcome is measured in total number of questions. Outcome raw scores for all tests are converted to scale points, where ≤ 7 scaled points indicate cognitive impairment. Results are reported for measured executive function, i.e. Verbal fluency, Cognitive flexibility and Problem solving.

The Behavior Rating Inventory of Executive Function–Adult (BRIEF-A) was used to measure Self-assessed executive functions. Higher score indicates that a participant experiences poorer executive function. T-scores are computed, and a cut-off score of 65 indicates impaired executive functions [21]. The Brief-A has shown strong internal consistency (0.93 to 0.96) and good validity differentiating between controls and substance use disorder [21, 22].

The diagnostic interview MINI [23] 7.0.1. was used to screen for the following psychiatric diagnoses: major depressive disorder, manic episode, hypomanic episode, panic disorder, agoraphobia, social anxiety, obsessive-compulsive disorder, posttraumatic stress disorder (PTSD), psychotic symptoms, anorexia nervosa, bulimia nervosa, binge-eating disorder, generalized anxiety disorder, antisocial personality disorder, and attention deficit hyperactivity disorder (ADHD). The MINI has displayed satisfactory reliability (kappa 0.45–0.75) and reasonable validity in distinguishing psychiatric from non-psychiatric issues among OMT patients [23, 24].

The Structured Clinical Interview for DSM-IV-R Personality Disorder (SCID II) self-report form (SCID-II screen), was used to assess symptoms and types of personality disorders [25]. The SCID-II has displayed demonstrates internal consistency coefficients ranging from acceptable to very good (0.35–0.83) and exhibits reasonable reliability (0.37–0.76) in substance use patients [26].

### Procedures

Data collection took place at a day care clinic for OMT within the public health care in western Sweden in 2022. The patients were enrolled through either referral or self-referral. Patients were informed about the study, and written consent was obtained during the first week at the day care unit. Exclusion criterion for participation was the inability to understand and speak Swedish, as participation required completing interviews, cognitive tests, and self-assessment forms in Swedish. Researchers and OMT clinic staff assessed which patients were too ill to participate (e.g., due to ongoing psychosis or severe withdrawal).

Data collection was initiated at admission and conducted on two separate occasions within three weeks to accommodate the cognitive demands of the tests. The first session, lasting 60 to 120 min, included a semi-structured interview (not included in this paper) prior to cognitive testing, which comprised three subtests from the D-KEFS. Participants then completed the BRIEF-A and SCID-II screening forms. The second session, lasting 30 to 60 min, consisted of the MINI structured interview.

Demographic information was collected at both sessions, and drug tests were routinely administered at the start of treatment by clinic staff, with results obtained by the researchers from patient records.

Patients were followed up after one year of treatment, with repeated cognitive testing, the BRIEF-A screening form, and drug tests to examine concurrent substance use.

The study protocol was approved by the Swedish Ethical Review Authority, DNR: 2020–03753, in accordance with the Declaration of Helsinki.

### Data processing and statistical analyses

SPSS version 28.0 was used for statistical analyses. The variable *duration of drug use* was computed by subtracting the age at onset of drug use from the participant’s current age. Descriptive statistics were calculated to provide means (*m*), standard deviations (*SD*), median (*Mdn*), percentages, and ranges of demographic data along with executive function, psychiatric, and substance use data. We present data primarily based on the results of parametric tests (independent t-test, paired samples t-test, Pearson correlation), but we have also examined the data using non-parametric tests (Mann-Whitney U, Spearman correlation, chi-square test). When there is no difference between the outcomes of the different testing methods, we report the results from the parametric tests. Cohen’s d (*d*) was used to examine effect size. In the supplementary section, tables are provided for several analyses in which we examined potential sex differences. Given that this is an exploratory study, we have set the significance level at *p* <.1 to minimize the risk of Type II errors. Hence, significant results should be interpreted with caution.

## Results

### Executive function

**Fig. 1:**
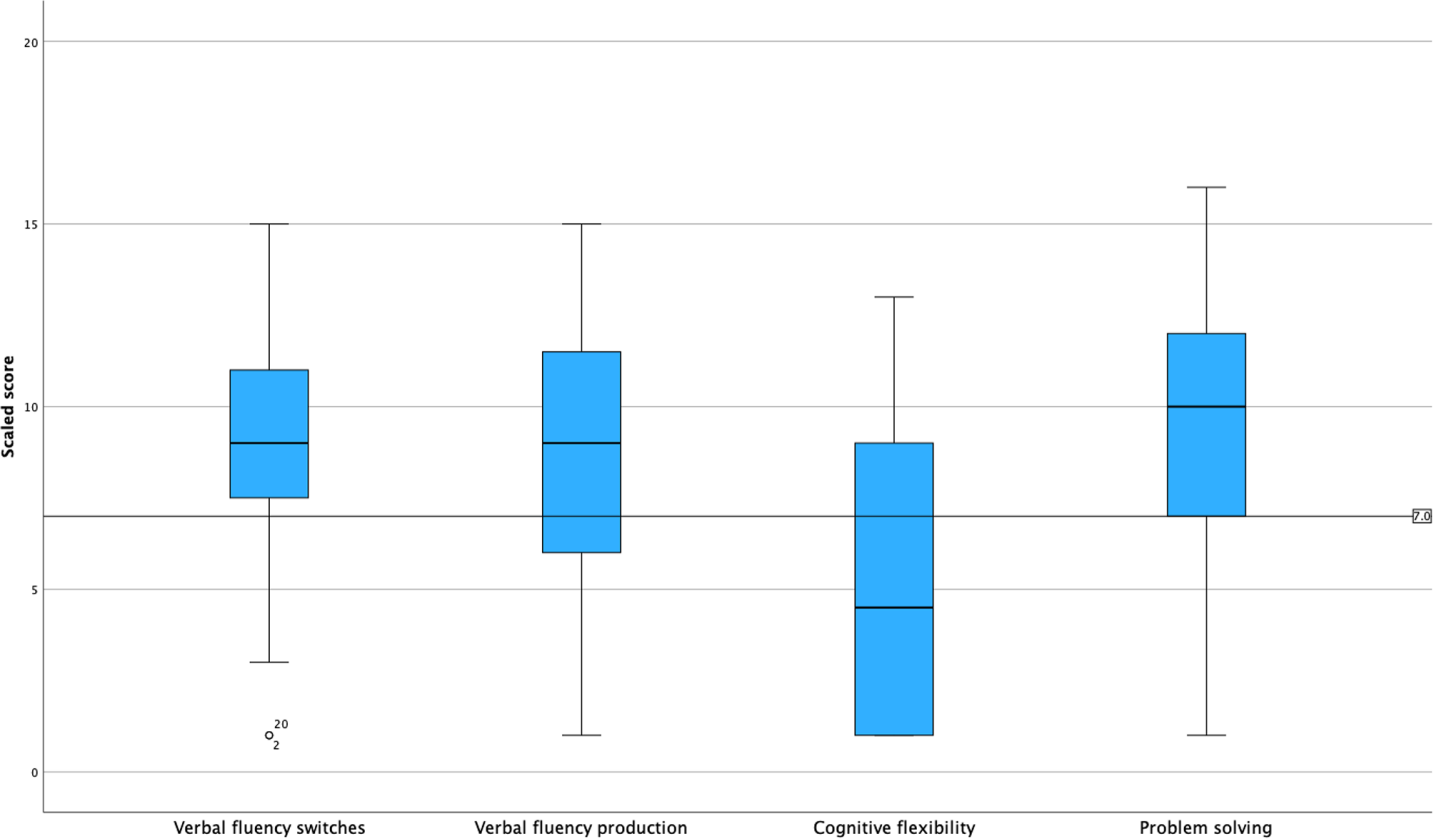
Box plots displaying the median, quartiles and the outliers of the different executive function tests (Verbal fluency switches, Verbal fluency production, Cognitive flexibility and Problem solving). The line inside the boxes denotes the median of the executive functions scales. Lines (whiskers) extend from the box to show the range of the rest of the data. The outliers are plotted individually. The horizontal line running through all the boxes marks the cutoff for impairment (< 7)

Forty-eight of the participants at admission completed the tests of executive function. The test results are summarized in the boxplot (Fig. 1). Means and standard deviations for the various tests are also presented in Table 2.

**Table 2:** Change in cognitive function between admission and follow-up. The table on the right also compares baseline data on the patients who have agreed to undergo cognitive testing at follow-up and the patients who have declined test follow-up

	Baseline total	Admission	One-year follow-up	d	Base-line data followed up	Base-line data declined/failed to appear to follow-up testing	d
Verbal fluency switching	9.19 (3.44) *n* = 48	8.83 (3.75) *n* = 24	10.42 (3.20)* *n* = 24	-0.48	8.83 (3.75) *n* = 24	9.54 (3.15) *n* = 24	0.21
Verbal fluency production	8.88 (3.70) *n* = 48	8.63 (3.72) *n* = 24	10.67 (3.10)* *n* = 24	-0.52	8.63 (3.76) *n* = 24	9.13 (3.73) *n* = 24	0.13
Cognitive flexibility	5.13 (4.01) *n* = 48	5.26 (4.09) *n* = 23	5.61 (4.34) *n* = 23	-0.12	5.26 (4.09) *n* = 23	5.00 (4.02) *n* = 25	-0.06
Problem solving	9.48 (3.44) *n* = 48	9.27 (3.38) *n* = 22	9.86 (2.98) *n* = 22	-0.13	9.27 (3.38) *n* = 22	9.65 (3.54) *n* = 26	0.11
Self-assessed executive function	134.48 (21.42) *n* = 42	129.80 (22.20) *n* = 15	121.60 (25.62) *n* = 15	0.476	129.80 (22.20) *n* = 15	137.07 (20.94) *n* = 27	0.34

* *p* <.05

*Verbal fluency*: Mean scaled score for correct switching between categories was 9.2 (SD = 3.4), where twelve of the participants (25%) exhibited impaired function. Mean scaled score for correct words was 8.8 (SD = 3.7), where fifteen of the participants (31%) exhibited impaired function. *Cognitive flexibility*: Mean scaled score for completion time was 5.1 (SD = 4.0), where 32 of the participants (67%) exhibited impaired function. *Problem solving*: Mean scaled score for number of questions used was 9.5 (SD = 3.4), where fourteen of the participants (29%) exhibited impaired function.

*Self-assessed executive function*: Forty-two participants filled in the self-assessment scale of executive function, (BRIEF-A). Mean was 66.0 (SD = 10.4). Twenty-seven out of 42 participants (64%) reported executive difficulties (data not shown in the figure).

No significant differences (using parametric and non-parametric tests) between male and female participants were detected on executive function and Self-assessed executive function at the initiation of treatment and at the one-year follow-up (data not shown).

### Psychiatric diagnosis and personality disorders

*MINI*: The prevalence of psychiatric diagnosis among 38 participants (the participants who were able to be diagnosed) are presented in Table 3. The average number of psychiatric diagnoses was 3.3 (SD = 2). *SCID-II screen*: The prevalence of self-rated personality disorders among 39 participants is presented in Table 3. The average number of self-rated personality disorders was 5.9 (SD = 2.4). (Sex stratified data on psychiatric diagnoses and self-rated personality disorders are shown in the supplementary section, Table 1).

**Table 3:** Number and percentage of participants (*n* = 38) who have reached the criteria for each diagnosis according to MINI. Number and percentage of participants (*n* = 39) who reached the criteria for each personality disorder according to SCID-II

Psychiatric diagnosis (MINI)	*n*	%	Personality disorder (SCID-II)	*n*	%
Depression	33	87	Borderline	33	85
ADHD	12	32	Antisocial	26	67
Panic disorder	12	32	Narcissistic	24	62
PTSD	11	29	Paranoid	22	56
Social anxiety	8	21	Compulsive	22	56
Agoraphobia	8	21	Depressive	21	54
OCD	7	18	Passive-aggressive	21	54
Generalized anxiety disorder	6	16	Schizotype	17	44
Manic episode	5	13	Phobic	17	44
Psychotic symptoms	4	11	Dependent	14	36
Hypomanic episode	1	3	Schizoid	10	26
Binge-eating disorder	0	0	Histrionic	4	10
Bulimia	0	0			
Anorexia nervosa	0	0			

### Drug use at admission and the follow-up

Ongoing drug use (assessed through saliva and blood samples) was monitored in 41 participants both at the initiation of treatment and after one year. All participants (100%) were using opiates/opioids at the start of treatment, at follow-up, illegal opiate use was still detected in six individuals (15%). Benzodiazepine use was the second most common drug at the beginning of treatment, with 22 participants (54%) using benzodiazepines. At the one-year follow-up, there were still 20 participants (49%) using benzodiazepines. Stimulants was used by 11 (27%) at the start of treatment. There were still 8 participants (20%) using stimulants at the follow-up. For further details on types of drugs used, see Table 4. The average number of drugs used at admission was 4.1 (SD = 2.3) with the range of 0–11. The total number of drugs used had changed significantly from admission to follow-up, with fewer drugs used overall at the follow-up, 1.9 (SD = 1.9) *t* [27] = 5.31, *p* <.001. The effect size was *d* = 0.81.

**Table 4:** Presence of the number of substances in blood testing at admission (*n* = 41) and during one-year follow-up (*n* = 41)

	Admission *n* (%) mean (sd)	One-year follow-up *n* (%) mean (sd)
Opioids	41 (100%)	6 (15%)
Benzodiazepines	22 (54%)	20 (49%)
Stimulants	11 (27%)	8 (20%)
Cannabis	3 (7%)	4 (10%)
Empathogens	2 (5%)	0 (0%)
Alcohol (*n* = 26)	8 (35%)	5 (22%)
Total number of present substances	4.1 (2.3)	1.9 (1.9)***

*** *p* <.001 *d*: 0.810

### Correlations

In Table 5, correlations among cognitive tests and Self-assessed executive function are shown. It was observed that several of the tests showed moderate correlations, yet none of the tests correlated with self-assessed executive (function). (Sex stratified data with correlations among cognitive tests and Self-assessed executive function are shown in the supplementary section, Tables 2 and 3).

**Table 5:** Correlations of executive functions and self-assessed executive function (*n* = 48)

	Verbal fluency switching	Verbal fluency production	Cognitive flexibility	Problem solving	Self-assessed executive function
Verbal fluency switching	1.00	0.853***	0.512***	0.468***	0.113
Verbal fluency production		1.00	0.456***	0.430***	0.177
Cognitive flexibility			1.00	0.443***	0.009
Problem solving				1.00	0.097
Self-assessed executive function					1.00

*** *p* <.001

In Table 6, correlations between executive function and current use of alcohol, benzodiazepines, and stimulants (the three most commonly detected drugs upon drug screening) are presented, along with the three most prevalent personality disorders and the three most common psychiatric diagnoses. Additionally, correlations between age of drug initiation, duration of drug use, and the number of drugs used at admission and the follow-up, are, reported. The results indicated that stimulants exhibited a negative correlation with Verbal fluency 1 (switching) *r*(45) = − 0.256, *p* <.1, a negative correlation with Verbal fluency 2 (production), *r*(45) = − 0.255, *p* <.1. and a negative correlation with Cognitive flexibility r(45) = − 0.281, *p* <.1.

**Table 6:** Correlations of executive functions, self-assessed executive function and substance use and psychiatric comorbidity

	Verbal fluency switching	Verbal fluency production	Cognitive flexibility	Problem solving	Self-assessed executive function
Alcohol use	− 0.062	− 0.101	− 0.231	− 0.005	0.111
Bensodiazepine use	− 0.098	− 0.084	− 0.200	− 0.126	0.060
Stimulant use	− 0.256*	− 0.255*	− 0.281*	− 0.053	0.221
Borderline	0.119	0.146	− 0.165	0.111	0.575***
Antisocial	0.143	0.153	0.055	0.286*	0.228
Narcissistic	− 0.222	− 0.046	− 0.272*	0.093	0.256
Depression	− 0.047	− 0.154	0.037	0.090	0.175
ADHD	− 0.012	− 0.011	0.008	0.136	0.209
Panic disorder	0.105	0.147	0.081	− 0.173	− 0.032
Debut of substance use	− 0.111	− 0.126	− 0.259*	− 0.107	− 0.379**
Age	-428**	-418**	-135	-210	− 0.226
Years of substance use	− 0.407**	-391**	− 0.048	− 0.165	− 0.129
Presence of substance use at admission (total number)	-224	-232	-251	-021	0.249
Presence of substance use at follow-up (total number)	0.098	0.111	0.247	0.167	0.220

* *p* <. 0.1

** *p* <.05

*** *p* <.001

Borderline personality disorder exhibited a significant positive tendency with Self-assessed executive function *r* [28] = 0.575, *p* <.001. Antisocial personality disorder exhibited a positive tendency with Problem solving *r* [28] = 0.286, *p* <.1. Narcissistic personality disorder exhibited a negative tendency with Cognitive flexibility *r* [28] = − 0.272, *p* <.1.

Debut of substance use exhibited a significant negative tendency with Cognitive flexibility r(47) = − 0.259, *p* <.1 and a significant negative correlation with Self-assessed executive function r [29] = − 0.379, *p* <.05. Age exhibited a significant negative correlation with Verbal fluency 1 (switches), r(47) = − 0.428, *p* <.001, and a significant negative correlation with Verbal fluency 2 (production), r(47) = − 0.418, *p* <.001. Years of substance use exhibited a significant negative correlation with Verbal fluency 1 (switches), r(47) = − 0.407, *p* <.001, and a significant negative correlation with Verbal fluency 2 (production), r(47) = − 0.391, *p* <.001. No other variables showed significant correlations or tendencies with executive function; see Table 6. (Sex-stratified data from these analyses are shown in the supplementary section, Tables 4 and 5. Some differences in trends between males and females were detected in these analyses. Due to the small sample size, it is difficult to draw any conclusions).

### Change in executive function from admission to one-year follow-up

Twenty-four participants completed the follow-up examination of executive function. Verbal fluency (verbal switching t [21] = -2.35, *p* =.028 and verbal production t [21] = -2.53, *p* =.019) improved significantly see Table 2. The effect size, as measured by Cohen’s d, was d = -0.48 and d = -0.52 indicating a moderate effect. No improvement nor decrease in performance was found in Cognitive flexibility, Problem solving and Self-assessed executive function.

Comparison with other participants showed no significant differences in initial executive function, see Table 2.

## Discussion

Our study aimed to examine the executive function in patients with opioid use disorder at the onset of treatment and at a one-year follow-up and its relationship with the most prevalent clinical factors within the patient group. Below, the main results are discussed and compared to findings from previous research.

Participants displayed severe difficulties of executive function in comparison to the normal population. In terms of Cognitive flexibility, its mean score was below the normal range, with 67% displaying impaired function. Notably 16 of the 43 participants (37%) received the lowest possible score, which means they did not complete the test within the prescribed time. Also, no participant scored higher than the normal area on the Trail Making Test (measuring *Cognitive flexibility*), indicating a curtailed function not found in the other test results. Strauss et al. [30] showed normal population completion time for Trail Making Test at 87 s among the lowest tenth percentile. The present study displayed an average completion time of 153 s for *all* participants (almost twice the normal time). Results are comparable to a study of participants with schizophrenia (the majority also had concomitant substance use syndrome) where almost half of the participants displayed the lowest possible score of Trail Making Test (more about this under limitations) [31]. The authors therefore suggested that the floor should be lowered for the test, through adjusted and sample-specific norms (for example, extended completion time would enable certain participants to complete the test and might point to poor motor speed as the contributing factor of test performance, indicating specific functional weakness). It is reasonable to assume that the present study is also affected by a so-called floor effect, indicating that instruments fail to distinguish those who have performed the worst and might conceal both specific weaknesses and statistical association between variables.

Mean values for Verbal fluency and Problem solving were within the normal area. However, the test results within the group showed a significant variation where 25 − 30% of the participants of the respective groups displayed impaired function, compared to 2.5% in a normal population [10].

*Self-assessed executive difficulties* were reported in 75% of the participants. The number of patients with self-assessed executive difficulties was higher than, for instance, a group of patients with traumatic brain injury traumatic brain injury [21]. The study’s findings are consistent with previous research, where the same instrument was used to evaluate self-reported executive difficulties among individuals with substance use disorder [22, 32].

All tests of executive functions correlated with each other but, interestingly, none of the test results correlated with Self-assessed executive function, suggesting that the two assessment methods investigate different aspects of executive function. It is important not to assume that cognitive testing provides more objective results than self-rating, because the participant’s rating is based on experiences in real life compared to the testing situation, which can be more artificial [22]. On the other hand, it is important to not dismiss testing as non-relevant to real life. Test results can indicate the gap between normal and impaired function of specific and general executive function that are non-distinguishable in everyday life.

The overall results of executive function are in line with previous research linking opioid addiction to impaired executive function [22, 33]. Scott et al. [5] reported a similar 31% impairment in Verbal fluency and a lesser 35% impairment in Cognitive flexibility among OMT patients.

To our knowledge no other study has used a structured interview with similar scope to screen for the most common psychiatric diagnosis at the start of OMT. Psychiatric comorbidity was prevalent, underscoring the need for thorough screening, particularly given the lower prevalence reported in other studies. For example, Scott et al. [5] reported a 37% prevalence of depressive disorder, compared to 85% in the present sample. Similarly, personality disorders were also common in this group. The screening part of SCID-II is designed to be over-inclusive, which may explain the extensive prevalence of personality disorders within the patient group. However, the high prevalence of personality disorder diagnoses in this patient group is in line with previous studies of OMT [34].

Polydrug use was prevalent with wide variation and comparable to both Öhlin et al., [26] report of an OMT sample, and to other substance use populations [35]. However, present sample displayed higher duration of drug use, on average 25 years, compared to Ersche et al. [36] who reported 10-year average among opiate users exhibiting cognitive impairment. Our results align with Monwell et al. [35], who highlighted general polydrug use in society and recommended cognitive support tools to aid treatment. However, in contrast to Loeber et al. [37] who found an association between polydrug use and executive function in OMT patients, our study did not display this correlation. Stimulants, but not alcohol or benzodiazepines, were associated with impaired executive function, specifically in Verbal fluency and Cognitive flexibility, suggesting a particularly detrimental impact on cognition. This is consistent with Ersche et al. [36] who reported greater impairment in amphetamine users than in opiate users. Notably, there was no correlation between Self-assessed executive function and stimulant use, indicating that patients may not perceive greater cognitive impairment than those without stimulant use.

The only demographic difference observed between sexes was the age of drug initiation, with females exhibiting a later onset. However, no other differences between male and females were detected regarding executive function. Instead, the strongest significant correlation was observed between age and the number of years of substance use and executive functions. However, this was limited to various aspects of Verbal fluency and did not extend to the other tested executive functions or to Self-assessed executive function. Ersche et al. [36] reported no effect of years of substance use on cognitive function, in contrast to our findings. The age of drug initiation also showed a negative correlation with both Cognitive flexibility and Self-assessed executive function. This finding suggests that a later onset of drug use is associated with better self-reported executive functioning, which is expected; however, it is also unexpectedly linked to poorer Cognitive flexibility. This in contrast to a study by Capella et al. [38] of how SUD patients, with an earlier onset of substance use (16 years or younger) had poorer clinical states, lower premorbid IQ, and deficits in EF. Our findings, in conjunction with previous research, demonstrate variable effects of substance use on executive function, underscoring the importance of localized monitoring of patient groups across diverse substances, usage patterns and demographic factors. This approach may have broad applicability across all types of addiction populations.

To our knowledge, no other study has investigated self-reported symptoms of personality disorders and their correlation with executive functioning in OUD patients. Greater symptoms of Borderline Personality Disorder, were linked to poorer self-reported executive function, but not to tested executive function. This suggest that OUD patients with symptoms of Borderline Personality Disorder may perceive greater executive difficulties than are evident in performance. In contrast, symptoms of Narcissistic Personality Disorder were associated with impairments in Cognitive flexibility. Symptoms of Antisocial Personality Disorder showed a positive correlation with problem-solving ability in the present sample, indicating an unexpected relationship between greater symptom severity and better functioning. This finding is surprising given the disorder’s association with deficits and treatment attrition [8, 11]. Further investigation with a larger sample and potentially different methodologies, such as experimental approaches, is necessary to explore the relationships in greater detail. Regarding other psychiatric diagnoses, there were no correlations found either between testing of executive function or Self-assessed executive function. A recent study by Marques-Arrico et al. [39] found that patients with both Substance Use Disorder (SUD) and Major Depressive Disorder performed worse on cognitive tasks and reported lower social functioning compared to those with SUD alone, indicating a link between objective and subjective performance. Research on Opioid Maintenance Treatment (OMT) patients has shown varying relationships between psychiatric comorbidities, such as depression, and executive function. Further research could explore objective and subjective performance in OMT patients with and without illicit substance use and psychiatric comorbidity.

The longitudinal findings indicate a moderate improvement in Verbal Fluency, including switching and productivity, while Cognitive flexibility and Problem solving remained unchanged. These results suggest that after one year of treatment, less complex functions like Verbal fluency may improve, while overall executive function and more complex skills may not. Consistent with prior mixed findings, our clinical sample—with minimal exclusion criteria—showed specific improvements but no general gain in executive functions, unlike samples with lower psychiatric comorbidity [18]. The limited evidence suggests the possibility of both improvement and persistent impaired executive function in OMT-patients with comorbidities. Clearly, more research in this area is needed due to the limited number of studies available.

### Clinical importance

Previous research vividly illustrates the vulnerability of this patient group, but mixed results may suggest the necessity for tailored local treatment approaches. Monitoring the aging patient population may be clinically crucial due to the diverse results of correlating factors observed in this study. Previous longitudinal studies use clinical samples but exclude participants with side use of substances which is a substantial portion of the OMT patient population [17, 18]. In this regard our finding of improved Verbal fluency is promising, particularly given the high comorbidity within the sample and the broader OMT population.

The results regarding correlates suggest the necessity for continuous monitoring of these clinically important factors. This may be crucial for attributing the appropriate level of care and support, as well as for implementing tailored treatment interventions. At a policy level, funding must be allocated to support care for this patient population, addressing both habilitation and rehabilitation needs.

Qualitative research syntheses highlight patients experience treatment barriers including insufficient knowledge of OMT patients among healthcare personnel and suboptimal communication between patients and staff. Patients also request help with many aspects of their lives, including everyday functioning in society [28]. These are areas where this study can provide valuable insights into patients’ levels of functioning. For example, housing support and other social resources, which play a critical role for patients, are often constrained by functional gaps and regulatory barriers, underscoring the importance of adequate assessment for goal setting.

Promising results from randomized controlled trials (RCTs) include a study that demonstrated computerized Working memory training in methadone maintenance patients led to improvements in certain aspects of working memory [40]. Additionally, digital intervention targeting executive function exhibited reduced craving and impulsivity among methadone maintenance patients [41]. As injectable buprenorphine offers a more flexible outpatient care regimen these findings highlight a new avenue for supporting patients where trained psychologists could utilize digital tools to assess and train executive functions, enabling remote monitoring and care.

### Limitations

As this study is exploratory, we employed a liberal significance threshold (*p* <.1) to examine relationships among variables and minimize the risk of Type II errors. Nonetheless, results should be interpreted with caution, and extensive conclusions should be avoided. Further research with a larger sample is essential to gain a more comprehensive understanding of these associations. Comparisons with prior research should also be made cautiously, considering the limitations in validity and generalizability. The small sample size restricts generalization, including for longitudinal findings. However, previous longitudinal studies also present comparative sample sizes [17, 18]. Other operationalizations and definitions of executive functions in research may limit comparisons. Measures of psychiatric comorbidity (MINI and SCID-II) can only be viewed as parallel problems and cannot be assessed as primary or secondary to substance use, limiting causal inferences. SCID-II is self-reported and not diagnosed personality disorder. On the other hand, D-KEFS provide a valid measure of executive function and BRIEF-A is recommended for self-rated executive functions [22, 32, 29]. The statistical floor effects mentioned earlier may obscure both correlates and deteriorated longitudinal results.

## Conclusion

Opioid maintenance treatment arose from Dole and Nyswander’s research [27] and has since clinically improved the prognosis of the opioid addicted. The prognosis in the current era of the global epidemic is not great, however, and new knowledge is needed regarding treatment risk factors such as executive function. The present study suggests considerable impairment of general cognitive functions, especially the results of Cognitive flexibility. Results show improved Verbal fluency after one year of OMT but not an overall improvement of executive function, indicating persistent impairment. High levels of other risk factors are prevalent, including psychiatric comorbidity and polysubstance use. Future research should track the aging population within this group, comparing outcomes with other addiction populations, especially as polysubstance use is prevalent. Aging further emphasizes the necessity of recruiting patients for treatment at the earliest possible stage. Our data echo the results of previous research and indicate the need of extensive care for OMT patients due to impaired executive functioning and heavy psychiatric comorbidity.

## Electronic supplementary material

Below is the link to the electronic supplementary material.

Supplementary Material 1

## Data Availability

The datasets used and/or analyzed during the current study are available from the corresponding author on reasonable request.

## Abbreviations

ADHD: Attention Deficit Hyperactivity Disorder
BRIEF-A: The Behavior Rating Inventory of Executive Function–Adult
D-KEFS: Delis-Kaplan Executive Function System
LC-HRMS: Liquid Chromatography–High Resolution Sass spectrometry
MINI: Mini International Neuropsychiatric Interview
OMT: Opioid Maintenance Treatment
OUD: Opioid Use Disorder
PTSD: Post Traumatic Stress Disorder
SCID-II: The Structured Clinical Interview for DSM-III-R Personality Disorder self-report form
TMT: Trail Making Test B
VFT: Verbal fluency Test
