# Supplementary material for: Executive function at baseline and follow-up in opioid maintenance patients and its relation to psychiatric comorbidity and substance use patterns
Source: BMC Psychiatry. 2025 Apr 17;25:396. doi: 10.1186/s12888-025-06524-w (PMC12007326; doi:10.1186/s12888-025-06524-w)
Supplement: Supplementary file 1 — Supplementary Material 1 [file 12888_2025_6524_MOESM1_ESM.docx]

**Complementary tables**

Table 1. Prevalence of psychiatric diagnoses and self-reported personality disorders, stratified by gender

| **Psychiatric diagnosis** | **Men *n* = 29** | **Women *n* = 9** | **Personality disorder** | **Men (n = 32)** | **Women (n = 7)** |
| --- | --- | --- | --- | --- | --- |
| Depression | 25 (86.2 %) | 8 (88.9 %) | Borderline | 27 (84.4 %) | 6 (85.7 %) |
| ADHD | 11 (37.9 %) | 1 (11.1 %) | Antisocial | 24 (75 %) | 2 (28.6 %) |
| Panic disorder | 8 (27.6 %) | 4 (44.4 %) | Narcissistic | 22 (68.8 %) | 2 (28.6 %) |
| PTSD | 7 (24.1 %) | 4 (44.4 %) | Paranoid | 20 (62.5 %) | 4 (57.1 %) |
| Social anxiety | 6 (20.7 %) | 2 (22.2 %) | Compulsive | 17 (53.1 %) | 5 (71.4 %) |
| Agoraphobia | 6 (20.7 %) | 2 (22.2 %) | Depressive | 17 (53.1 %) | 4 (57.1 %) |
| OCD | 6 (20.7 %) | 1 (11.1 %) | Passive-aggressive | 18 (56.3 %) | 3 (42.9 %) |
| Generalized anxiety disorder | 4 (13.8 %) | 2 (22.2 %) | Schizotype | 14 (43.8 %) | 3 (42.9 %) |
| Manic episode | 3 (10.3%) | 2 (22.2 %) | Phobic | 13 (40.6 %) | 4 (57.1 %) |
| Psychotic symptoms | 3 (10.3 %) | 1 (11.1 %) | Independent | 12 (37.5 %) | 2 (28.6 %) |
| Hypomanic episode | 0 (0 %) | 1 (11.1 %) | Schizoid | 7 (21.9 %) | 3 (42.9 %) |
| Binge-eating disorder | 0 (0 %) | 0 (0 %) | Histrionic | 4 (12.5 %) | 0 (0 %) |
| Bulimia | 0 (0 %) | 0 (0 %) |  |  |  |
| Anorexia nervosa | 0 (0 %) | 0 (0 %) |  |  |  |
|  |  |  |  |  |  |

**Table 2.** Correlations of executive functions and self-assessed executive function in women (*n* = 12)

|  | **Verbal fluency switching** | **Verbal fluency production** | **Cognitive flexibility** | **Problem solving** | **Self-assessed executive function** |
| --- | --- | --- | --- | --- | --- |
| Verbal fluency switching | 1.00 | .785** | .451 | .377 | -.027 |
| Verbal fluency production |  | 1.00 | .580* | .327 | .171 |
| Cognitive flexibility |  |  | 1.00 | .422 | -.312 |
| Problem solving |  |  |  | 1.00 | .085 |
| Self-assessed executive function |  |  |  |  | 1.00 |

*** *p* < .001

**Table 3.** Correlations of executive functions and self-assessed executive function in men (*n* = 36)

|  | **Verbal fluency switching** | **Verbal fluency production** | **Cognitive flexibility** | **Problem solving** | **Self-assessed executive function** |
| --- | --- | --- | --- | --- | --- |
| Verbal fluency switching | 1.00 | .861** | .538** | .485** | .223 |
| Verbal fluency production |  | 1.00 | .448** | .450** | .256 |
| Cognitive flexibility |  |  | 1.00 | .450** | .086 |
| Problem solving |  |  |  | 1.00 | .325 |
| Self-assessed executive function |  |  |  |  | 1.00 |

*** *p* < .001

**Table 4** Correlations of executive functions. self-assessed executive function. substance use and psychiatric comorbidity in women

|  | **Verbal fluency switching** | **Verbal fluency production** | **Cognitive flexibility** | **Problem solving** | **Self-assessed executive function** |
| --- | --- | --- | --- | --- | --- |
| Alcohol use | -.508* | -497 | -.371 | -.214 | -226 |
| Bensodiazepine use | -.372 | -.226 | .005 | .000 | .345 |
| Stimulant use | - | - | - | - | - |
| Borderline | .200 | .055 | -.205 | -.142 | .846** |
| Antisocial | -.042 | -.045 | -.126 | -.541 | .026 |
| Narcissistic | .091 | .011 | -.190 | -.541 | .623 |
| Depression | .056 | .175 | .343 | .158 | .713 |
| ADHD | .783** | .675** | .481 | .716** | - |
| Panic disorder | -.354 | .094 | .109 | -.607* | .161 |
| Debut of substance use | -.367 | -.296 | -.163 | -.220 | -.466 |
| Age | -581** | -259 | -179 | -558* | .008 |
| Years of substance use | -.568* | -215 | -.161 | -.584** | .156 |
| Presence of substance use at admission (total number) | -.215 | -361 | .120 | .063 | -.314 |
| Presence of substance use at follow-up (total number) | .218 | -.150 | .307 | .177 | -.210 |

**Table 5** Correlations of executive functions. self-assessed executive function and substance use and psychiatric comorbidity in men

|  | **Verbal fluency switching** | **Verbal fluency production** | **Cognitive flexibility** | **Problem solving** | **Self-assessed executive function** |
| --- | --- | --- | --- | --- | --- |
| Alcohol use | .040 | -.004 | -.199 | .056 | .241 |
| Bensodiazepine use | -.079 | -.093 | -.266 | -.178 | .055 |
| Stimulant use | -.224 | -.218 | -.315* | .026 | .286 |
| Borderline | .117 | .177 | -.154 | .188 | .461** |
| Antisocial | .168 | .173 | .011 | .316* | .276 |
| Narcissistic | -.291 | -.071 | -.398** | .114 | .155 |
| Depression | -.063 | -.231 | -.031 | .075 | .014 |
| ADHD | -.107 | -.096 | -.119 | -.026 | .230 |
| Panic disorder | .202 | .151 | .107 | .022 | -.076 |
| Debut of substance use | -.105 | -.142 | -.276 | -.091 | -.339* |
| Age | -395** | -440*** | -129 | -123 | -.313* |
| Years of substance use | -.367** | -405** | -.032 | -.067 | -.230 |
| Presence of substance use at admission (total number) | -211 | -188 | -336** | -033 | .366** |
| Presence of substance use at follow-up (total number) | .079 | .161 | .233 | .168 | .355 |

**p* <.01

***p* <.05

****p* <.01
